# Alzheimer’s disease polygenic risk score as a predictor of conversion from mild-cognitive impairment

**DOI:** 10.1038/s41398-019-0485-7

**Published:** 2019-05-24

**Authors:** Sultan Chaudhury, Keeley J. Brookes, Tulsi Patel, Abigail Fallows, Tamar Guetta-Baranes, James C. Turton, Rita Guerreiro, Jose Bras, John Hardy, Paul T. Francis, Rebecca Croucher, Clive Holmes, Kevin Morgan, A. J. Thomas

**Affiliations:** 1 Human Genetics Group, University of Nottingham, Nottingham, UK; 2 UK Dementia Research Institute at University College London and ION Department of Neurodegenerative Disease, London, UK; 3 Brains for Dementia Research Resource, Wolfson CARD, King’s College London, London, UK; 4 Faculty of Medicine, University of Southampton, Southampton, UK; 5 Institute of Neuroscience Biomedical Research Building Campus for Ageing and Vitality Newcastle University, Newcastle upon Tyne, NE4 5PL UK

**Keywords:** Clinical genetics, Molecular neuroscience

## Abstract

Mild-cognitive impairment (MCI) occurs in up to one-fifth of individuals over the age of 65, with approximately a third of MCI individuals converting to dementia in later life. There is a growing necessity for early identification for those at risk of dementia as pathological processes begin decades before onset of symptoms. A cohort of 122 individuals diagnosed with MCI and followed up for a 36-month period for conversion to late-onset Alzheimer’s disease (LOAD) were genotyped on the NeuroChip array along with pathologically confirmed cases of LOAD and cognitively normal controls. Polygenic risk scores (PRS) for each individual were generated using PRSice-2, derived from summary statistics produced from the International Genomics of Alzheimer’s Disease Project (IGAP) genome-wide association study. Predictability models for LOAD were developed incorporating the PRS with *APOE* SNPs (rs7412 and rs429358), age and gender. This model was subsequently applied to the MCI cohort to determine whether it could be used to predict conversion from MCI to LOAD. The PRS model for LOAD using area under the precision-recall curve (AUPRC) calculated a predictability for LOAD of 82.5%. When applied to the MCI cohort predictability for conversion from MCI to LOAD was 61.0%. Increases in average PRS scores across diagnosis group were observed with one-way ANOVA suggesting significant differences in PRS between the groups (*p* < 0.0001). This analysis suggests that the PRS model for LOAD can be used to identify individuals with MCI at risk of conversion to LOAD.

## Introduction

The genetic contribution to late-onset Alzheimer’s disease (LOAD) is now well established, with heritability estimates ranging from 58 to 79%^1^. The *APOE* gene located on Chromosome 19 encodes the Apolipoprotein E protein. This gene represents the largest genetic risk factor for LOAD to date, with genetic variation producing three isoforms: ε2 (protective), ε3 (neutral and most common form) and ε4, which is associated with increased risk for LOAD. Further to this a number of both common and rare genetic risk factors have been identified in recent years from genome-wide association studies (GWAS) and next-generation sequencing efforts^2–5^. Despite this increase in our knowledge of genetic associations these do not account for the entire heritability of the LOAD phenotype. Although other factors such as epistasis and epigenetics might contribute, it is becoming accepted that far more genes/polymorphisms with much smaller effect sizes are involved in complex diseases than previously envisaged.

Genetic risk calculation studies have typically used only variants identified by GWAS to try and predict LOAD phenotype^6–12^, mild- cognitive impairment (MCI) conversion to LOAD^13,14^, hippocampal cortical thickness^15,16^, hippocampal volume^17^, cerebrospinal fluid biomarkers^18^ and plasma inflammatory biomarkers^19^.

The development of polygenic risk score (PRS) analysis now allows for the sum of genetic risk from the entire genome to be accounted for, weighted by the effect size estimates attained from established GWAS data, rather than selecting a few specific associated single-nucleotide polymorphism (SNP) markers^20^. The study by Escott-Price et al.^21^ demonstrated that the predictability of LOAD from APOE isoform and contribution of the LOAD GWAS SNPs was improved by incorporating further variants from across the genome into their PRS model. The addition of other predictors such as gender and age into the model resulted in a final predictive ability of 78.2%. Several other studies have also demonstrated that this form of analysis can differentiate between controls and LOAD cases with similarly high accuracy^22,23^.

The pathological features of LOAD have been found to begin decades before the onset of symptoms^24–26^. Therefore, the early detection of those likely to be at risk for LOAD could increase effectiveness of treatments preventing further damage from occurring^25,26^. MCI is diagnosed in up to one-fifth of individuals over the age of 65 and is considered a prodrome of dementia^25^. An estimated one-third of those diagnosed with MCI will go on to develop LOAD over time^27^. Consequently, understanding genetic risk factors within LOAD pathogenic mechanisms can improve detection and promote treatment before the pathogenic state arises^24,26,28^. A therapeutic intervention at the MCI stage may provide an opportunity to prevent or delay conversion to LOAD^13,29^. Therefore, there is a need to investigate if MCI to LOAD conversion can be predicted using PRS analysis.

In this study, we developed a PRS model to predict LOAD diagnosis in the brains for dementia research (BDR) cohort^30^. This model was then applied to a longitudinal sample of individuals with MCI from the Southampton inflammation, cognition and stress (ICOS) study, to see if the model could predict those individuals who converted from MCI to LOAD.

## Materials and methods

### Samples

The BDR resource has recruited patients with dementia as well as cognitively healthy controls; post-mortem pathology was used to confirm and classify disease status^31^. This cohort consists of 302 LOAD cases and 137 controls, with no significant differences in the age at death and percentage of females between the cases and controls. The number of ε4 carriers was significantly higher amongst cases (*p* < 0.001; Table 1) as expected.

**Table 1:** Demographics of each group genotyped

Cohort	Group	*N*	Age	Females (%)	*APOE* ε4+ (%)	*APOE* ε4ε4 (%)
BDR	LOAD cases	302	83.0	146 (48.3)	196 (64.9)	39 (12.9)
	Controls	137	84.0	68 (49.6)	49 (40.1)	2 (1.5)
ICOS	MCI Non-converters	73	76.0	21 (28.8)	31 (42.5)	4 (5.5)
	MCI converters	49	79.0	26 (53.1)	23 (46.9)	4 (8.2)

The late-onset Alzheimer’s disease (LOAD) cases and controls were recruited from the brains for dementia research (BDR) resource. The individuals with mild-cognitive impairment (MCI) were recruited from a single study in Southampton, UK; conversion to LOAD was identified after 36-month follow-up. LOAD cases were shown to harbour more *APOE* ε4+ individual than controls (*p* < 0.001), but no significant differences were observed between the proportion of females or age at death. MCI converters were shown to have a significantly higher proportion of females in comparison to the non-converters (*p* = 0.008), with no significant differences observed for age or *APOE* ε4+ carriers.

Individuals with MCI were recruited from the ICOS study in Southampton, UK. Diagnoses were made using the Petersen criteria for amnestic MCI^32^. The study followed-up individuals over a 36-month period (until October 2017) to identify those who converted to a LOAD (‘converters’ *n* = 49), and those who remained MCI (‘non-converters’ *n* = 73). Age at recruitment and presence of at least one *APOE* ε4 allele was not found to be significantly different between the converters and non-converters; although the converter group was found to have a significantly higher proportion of females (*p* = 0.008; Table 1).

### Genotyping

DNA extraction was performed using a standard phenol chloroform method on either 2 ml of blood (MCI) or 100 mg of brain tissue (BDR). DNA quality was assessed using the Agilent 2200 TapeStation DNA integrity number (DIN; average DIN = 8.95) and quantified using Nanodrop 3300 spectrometry. All samples were collected with informed consent as governed by local guidelines at the point of collection; experimental procedures were approved by local ethics committees - Nottingham Research Ethics Committee 2 (REC reference 04/Q2404/130); London City and East NRES (REC reference 08/H0704/128 + 5), and completed in accordance with approved guidelines.

Genotyping was performed on the customised NeuroChip array^33^. Clustering was completed with the assistance of a cluster file provided by Blauwendraat and colleagues (2017) and the dataset was aligned to the GRCh37/hg19 assembly using files provided by Rayner, W (Personal correspondence, Nov 2017). Quality control of the raw data was completed using GenomeStudio v2 and PLINK v1.9^34^, with samples removed based on a call rate less than 90%, gender mismatch and deviation from European population parameters. SNPs were removed based on a call rate less than 95%, genotype frequencies significantly out of Hardy–Weinberg equilibrium with a Bonferroni corrected *p* value threshold (*p* = 1.03 × 10^−^^7^) and excess heterozygosity (±3 standard deviations from mean). SNPs where the minor allele was observed in less than 3 individuals were also removed.

The *APOE* SNPs rs7412 and rs429358 (which determine the isoform) were genotyped with TaqMan assays using standard protocols. At least one *APOE* ε4 allele was harboured by 70.9% of LOAD cases, 46.9% of MCI converters, 42.9% of MCI non-converters and 40.1% of controls (Table 1).

### Polygenic-risk score generation and predictability modelling

The *APOE* gene is the largest known risk factor for LOAD, with the region surrounding the locus displaying levels of strong linkage disequilibrium (LD). Therefore, SNPs which fell within the 500 kb region (chr19:45,160,844-45,660,844; GRCh37/hg19 assembly) surrounding the *APOE* gene were excluded from the dataset. The genotypes for the *APOE* ε status SNPs (rs7412 and rs429358) were then reintroduced to the dataset to ensure genetic risk from *APOE* was captured.

Samples from the 1000 Genomes Project Consortium (1000G; *n* = 2504) were compared ancestrally to the BDR and MCI samples using common ancestry informative markers. Principal component analysis was carried out in PLINK to verify all NeuroChip-genotyped samples (*n* = 561) fell within the cluster of European descent (*n* = 503) to guard against population stratification.

Data from the 1000G samples were also used to calculate LD structure. The clumping algorithm in PRSice-2^20^ was set to identify any SNPs within 250 kb in LD with an *r*^2^ threshold greater than 0.1 and ‘clump’ them together to be represented by most significantly associated SNP within each LD block, denoted the index SNP.

PRSice-2 was utilised to generate PRS for LOAD cases and cognitively healthy controls, using summary statistic data from the International Genomics of Alzheimer’s Disease Project (IGAP) genome-wide association study^35^. Significance values and effect sizes from the IGAP cohort were used as the base dataset to generate the best PRS model which was then applied to the BDR and MCI target datasets. Briefly, the PRS for each individual in the target dataset is generated from a summation of effect sizes from all the SNPs included in the best model. The best model was derived from testing the inclusion of SNPs (19–73,058 SNPs) from a range of *p* value thresholds in the base dataset (10^−6^ to 1), to see which threshold gave the largest Nagelkerke’s *R*^2^ value. These SNPs were then used to generate PRS for each individual in both the BDR and MCI cohorts.

The significance of differences in mean PRS between the four groups (controls, MCI non-converters, MCI converters and LOAD) was tested using one-way ANOVA with post hoc Tukey in SPSS v24. Spearman’s correlation analysis was also conducted by ranking the diagnosis groups from 0 (control), 1 (non-converters), 2 (converters) to 3 (LOAD).

Predictive ability of *APOE* alone and *APOE* with the PRS for identifying LOAD cases and converters in each cohort was calculated using Area Under the precision-recall Curve (AUPRC) in R using the ‘PRROC’ package^36^ (R Core Team, 2013). AUPRC was calculated to identify predictive ability, values range from 0 to 100%, where 0% is random classification and 100% is perfect classification^37^.

The effect of non-genetic predictors (age and gender) were estimated in SPSS, and were included with PRS in logistic regression analysis to enable incorporation into the predictive model.

LOAD cases and controls were distributed into deciles based on the range of probability values which accounted for PRS with *APOE*, gender and age at death. The proportions of LOAD cases which fell into each decile were calculated and are depicted alongside the proportion of controls within the same decile. This was also conducted for the proportions of MCI converters and non-converters within each decile distributed by PRS including *APOE*, gender and age at recruitment.

## Results and discussion

European ancestry of the BDR and MCI samples was confirmed with principal component analysis, as all samples clustered accordingly with European samples from the 1000G dataset. A plot of the first two principal components is presented in Supplementary Fig. 1. With confirmation that the BDR and MCI target datasets were or the same ethnic decent as the IGAP base dataset, analysis could proceed.

PRSice-2 derived Nagelkerke’s *R*^2^ values for a range of SNP *p* value thresholds from 1 × 10^−6^ to 1 in the IGAP base dataset were used to determine the best significance threshold for inclusion of SNPs required to distinguish between LOAD cases and controls. There was a total of 73,056 SNPs were found in both the IGAP base dataset and NeuroChip target datasets after LD based clumping (*r*^2^ > 0.1; kb = 250). The largest Nagelkerke’s *R*^2^ value generated was 0.138, suggesting the inclusion of all SNPs at the *p* value threshold of 1.07 × 10^−^^4^ into the PRS model plus the two *APOE* SNPs (total 167 SNPs; Supplementary Table 1). This model was then applied to both the BDR and MCI cohorts.

**Fig. 1:**
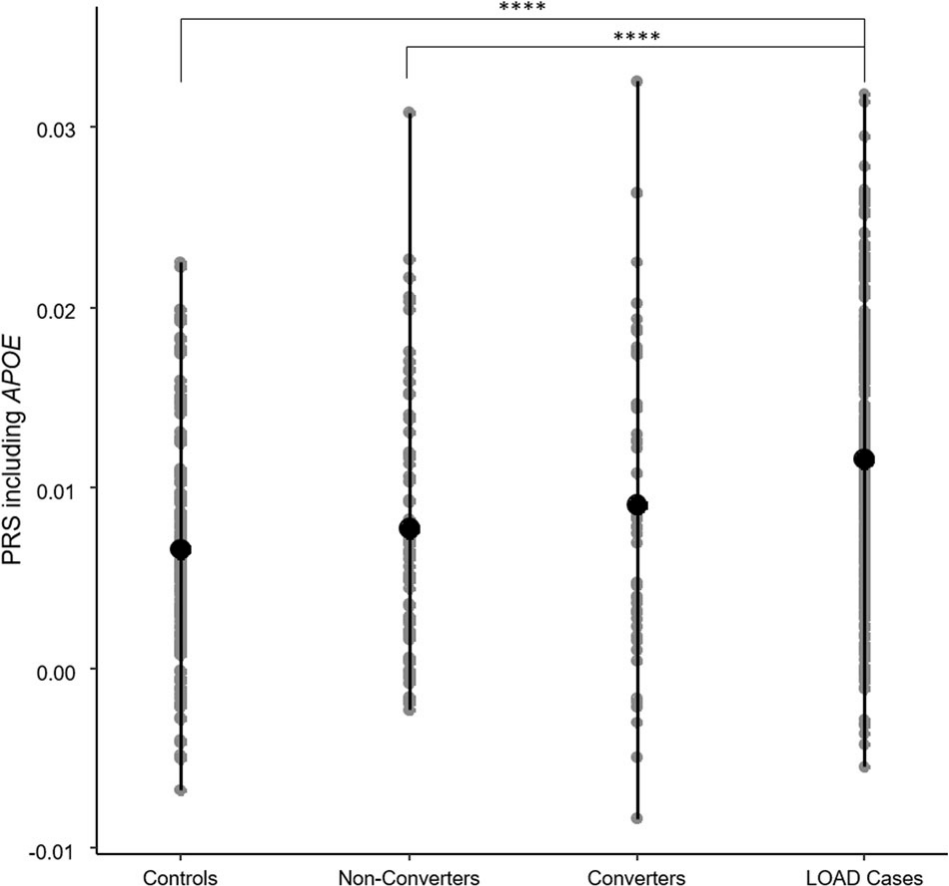
Distribution of polygenic risk score (PRS), including *APOE* SNPs (*N* = 167) amongst late-onset Alzheimer’s disease (LOAD) cases, converters and non-converters from mild-cognitive impairment (MCI), and controls. The range of scores for individuals within each group are described in the figure (grey circles) with the average PRS for each group indicated by the black circle. Significant differences were observed with one-way ANOVA across all four groups (*p* < 0.0001), with post hoc Tukey indicating significance between pairwise comparisons indicated with ****(*p* < 0.0001)

One-way ANOVA analysis of mean PRS scores between diagnosis groups, suggested an overall significance in PRS score (*p* < 0.0001). Pairwise post hoc Tukey analysis of the groups showed that the mean PRS of the BDR LOAD samples were found to be significantly higher than that of the controls (*p* < 0.0001 post hoc Tukey), likewise the mean PRS for the MCI converters was found to be higher than that of MCI non-converters, however this was not significant (Fig. 1). Post hoc Tukey also identified significant differences in mean PRS between non-converters and LOAD samples (*p* < 0.0001).

Predictability of the LOAD phenotype with PRS, including *APOE* with gender and age at death incorporated into the model, produced a final AUPRC for predictability for LOAD of 82.5%. The *APOE* genotypes alone were found to have a predictability of 81.8%. When applied to the MCI cohort for predictability of conversion with age at recruitment in the study instead of age at death, the full model produced a predictability of 61.0% for conversion, with *APOE* alone showing 43.8% predictability. This demonstrates the utility of the PRS to discern conversion from MCI to LOAD.

**Fig. 2:**
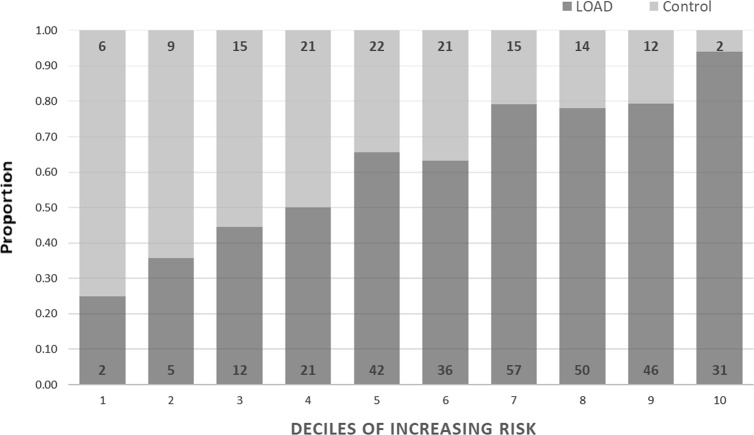
Proportion of late-onset Alzheimer’s disease (LOAD) cases and cognitively healthy controls in each decile using the best predictive model. Individual probabilities were generated using PRSice-2. The polygenic risk scores (PRS) including *APOE* and covariates for gender and age at death were used to distribute individuals into deciles. The dark bars represent the proportion of LOAD cases which fall into each decile and the light bars represent the proportion of controls. The number of LOAD cases and controls who fall within each decile are indicted in each block

**Fig. 3:**
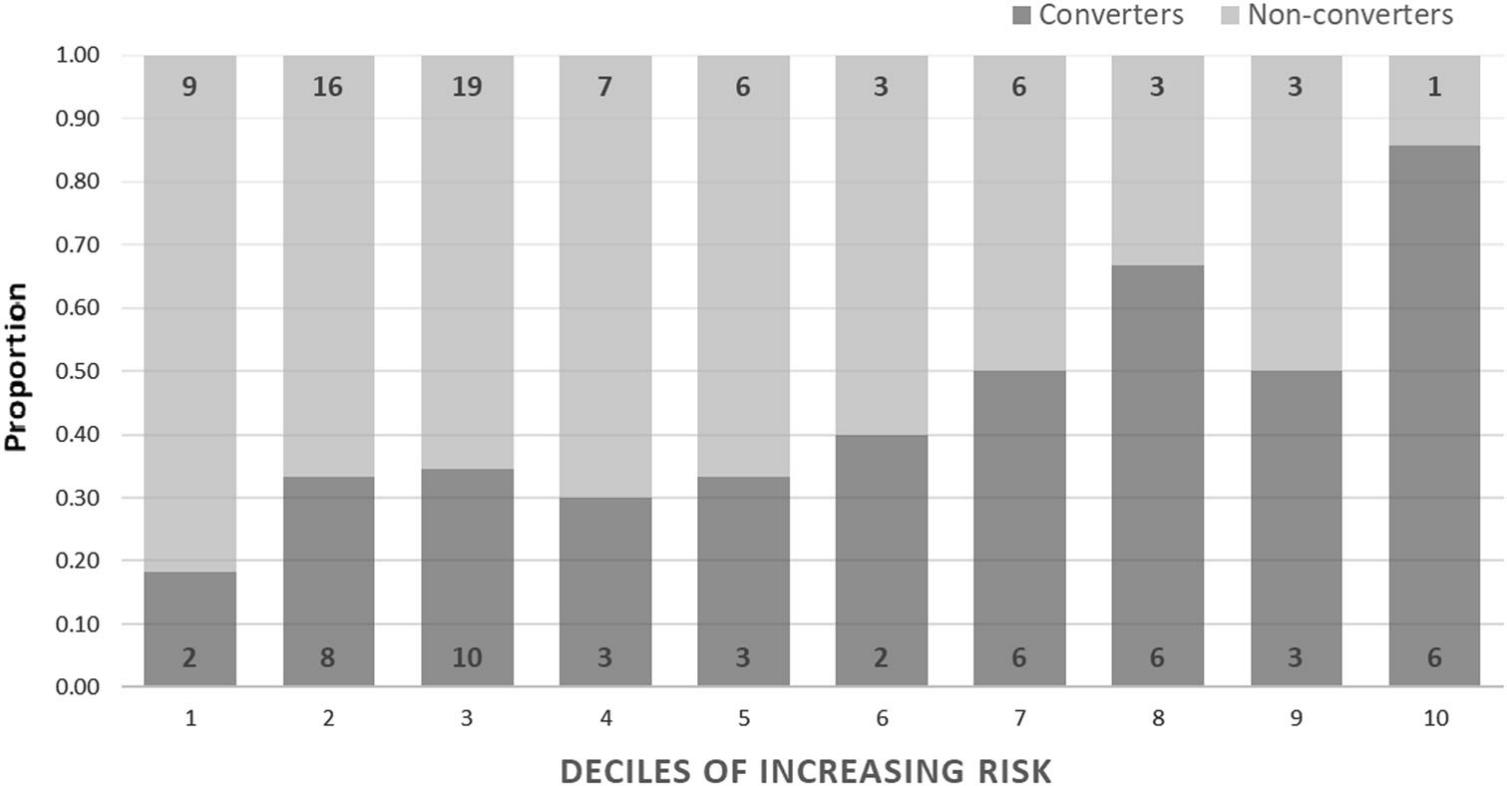
Proportion of mild cognitive impairment (MCI) non-converters and those who converted to late-onset Alzheimer’s disease. Individual probabilities for the best predictive model were generated from polygenic risk scores (PRS) including *APOE* and covariates for gender and age at recruitment to distribute individuals into deciles. The dark bars represent the proportion of converting MCIs in each decile and the light bars represent the proportion of non-converting MCIs. The number of converters and non-converters who fall within each decile are listed

Samples for the BDR and MCI cohorts were separately partitioned into deciles of increasing disease risk based on PRS, including *APOE* genotypes, gender and age; the proportion of LOAD cases and controls which fell into each decile are depicted in Fig. 2. As of December 2017, 49 individuals diagnosed with MCI had converted to LOAD (39.3%); the proportion of converters and non-converters which fell into each decile are also given in Fig. 3. Both figures show the proportion of LOAD/MCI converters increases with increasing PRS.

PRS is increasingly being used in genetic analyses to determine predictability of complex disease. In this study, cognitively healthy controls and individuals with LOAD or MCI were genotyped on the NeuroChip array^33^. PRS were generated from IGAP summary statistics and use to calculate risk scores for LOAD cases and controls from the BDR and MCI samples from the Southampton ICOS cohort. The best predictive model (82.5%) to distinguish LOAD cases from controls generated in the BDR cohort was found to be able to predict LOAD converters in the MCI cohort with an accuracy of 61.0%.

The difference between mean PRS of controls, non-converters, converters and LOAD cases was significant with one-way ANOVA, however, pairwise significance (post hoc Tukey) was only observed between LOAD cases and controls and LOAD cases and MCI non-converters. The distribution profile in Fig. 1 and Spearman’s rank order correlation (*r* = 0.408; *p* = 0.01) confirms an increased PRS with disease status from control through non-converters, converters to LOAD. The lack of a significant difference in average PRS between the MCI non-converters and converters is mostly like due to the small sample sizes, and it is expected that with increased numbers in each MCI group the mean PRS will move towards controls and LOAD cases, respectively. This hypothesis is supported by several studies suggesting that possession of LOAD-risk alleles are associated with faster cognitive decline, MCI and conversion from MCI to LOAD^13,38–40^, with one study concluding that carrying 6 or more of the non-*APOE* LOAD risk alleles rapidly increases conversion from MCI to LOAD^14^.

The resulting risk model, incorporating the *APOE* SNPs, 165 non-*APOE* SNPs, gender and age successfully predicted LOAD cases from controls with 82.5% accuracy which is a similar value to previous studies^21–23,25^.

As observed in previous PRS analyses^41^ there are a number of controls with high risk scores and cases of LOAD individuals with a low-risk scores (Fig. 2). It is possible that in these individuals, lifestyle and environmental factors could be determining the disease presence independent of, or interacting with genetic factors. Controls with higher PRS might represent individuals who would have developed disease had they lived longer as the average age at death of controls in the highest decile was significantly lower (73.5 ± 7.8 years) compared with the lowest decile (86.3 ± 5.0 years; *p* = 0.032). It is also possible that these individuals harbour unknown protective factors which may reduce disease risk.

Further study highlighted significantly later age at death in LOAD cases with the lowest risk compared to LOAD cases with the greatest risk (average age at death of LOAD in decile 1 = 95.0 ± 11.3; average age at death of LOAD in decile 10 = 81.1 ± 6.4; *p* = 0.007); this aligns with previous studies identifying later age at onset in cases with low PRS and increased prevalence with age;^42,43^. This could indicate that genetic load is associated with onset and/or severity of disease.

Predictive ability for LOAD conversion from MCI (61.0%) was less than between controls and LOAD (82.5%). This lower predictive ability is likely due to the smaller sample size of the MCI cohort, furthermore the ICOS cohort is a longitudinal study, and therefore unlike the BDR cohort which have the diagnoses post-mortem verified, the participants are still being clinically and cognitively assessed. It is possible that as the study progresses further individuals in this study will convert and influence the accuracy of the model.

The ability of the LOAD PRS model to predict to some degree the conversion of MCI to LOAD, highlights a possible genetic basis for conversion. Whereas non-converters have a genetic risk similar to that of controls, they may still harbour some genetic variations associated with LOAD which leads to the MCI phenotype though those with the lowest scores may never convert to LOAD. Conversely, the MCI subjects that did convert to LOAD have a PRS that is not significantly different to LOAD cases, which reinforces the idea that MCI can be seen as a prodromal state of LOAD and that by using approaches such as we describe, those at risk of developing LOAD can potentially be identified before the onset of LOAD symptoms and would be the best candidates to evaluate emerging therapeutic approaches.

## Supplementary information

Supplemental Figure 1

Supplemental Table 1
