# Correction: Alzheimer’s disease polygenic risk score as a predictor of conversion from mild-cognitive impairment

**DOI:** 10.1038/s41398-019-0503-9

**Published:** 2019-06-11

**Authors:** Sultan Chaudhury, Keeley J. Brookes, Tulsi Patel, Abigail Fallows, Tamar Guetta-Baranes, James C. Turton, Rita Guerreiro, Jose Bras, John Hardy, Paul T. Francis, Rebecca Croucher, Clive Holmes, Kevin Morgan

**Affiliations:** 1 Human Genetics Group, University of Nottingham, Nottingham, UK; 2 UK Dementia Research Institute at University College London and ION Department of Neurodegenerative Disease, London, UK; 3 Brains for Dementia Research Resource, Wolfson CARD, King’s College London, London, UK; 4 Faculty of Medicine, University of Southampton, Southampton, UK

**Keywords:** Clinical genetics, Molecular neuroscience

**Correction to:** **Translational Psychiatry**

10.1038/s41398-019-0485-7 published online 24 May 2019

In the original Article, Professor Alan Thomas was not acknowledged as an author. This has been updated in both the HTML and PDF versions of this Article.

