# Supplementary figures and images for: Alzheimer’s disease polygenic risk score as a predictor of conversion from mild-cognitive impairment
Source: Transl Psychiatry. 2019 May 24;9:154. doi: 10.1038/s41398-019-0485-7 (PMC6534556; doi:10.1038/s41398-019-0485-7)

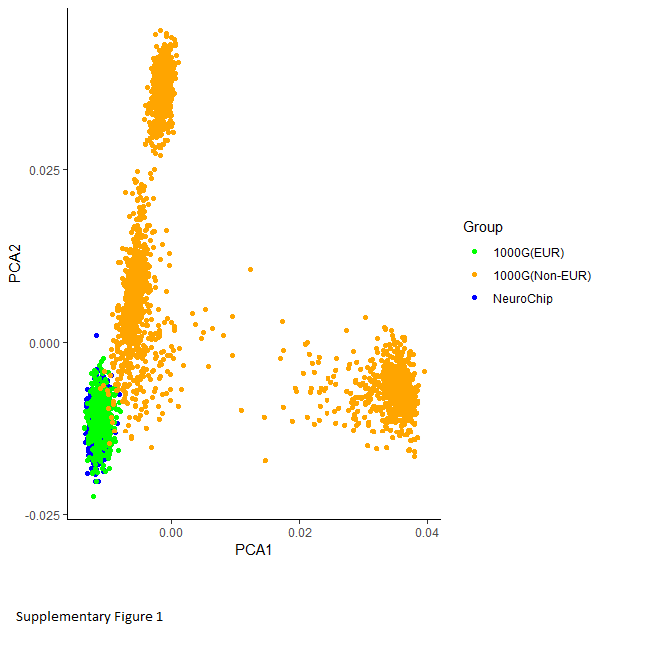

Supplement: Supplementary file 1 — Supplemental Figure 1 [file 41398_2019_485_MOESM1_ESM.tif]
